# Supplementary material for: Tertiary lymphoid structures critical for prognosis in endometrial cancer patients
Source: Nat Commun. 2022 Mar 16;13:1373. doi: 10.1038/s41467-022-29040-x (PMC8927106; doi:10.1038/s41467-022-29040-x)
Supplement: Supplementary file 1 — Supplementary Information [file 41467_2022_29040_MOESM1_ESM.pdf]

Supplementary figures

Supplementary Figure 1. Single cell RNA sequencing of tumor-infiltrating B-cells per patient

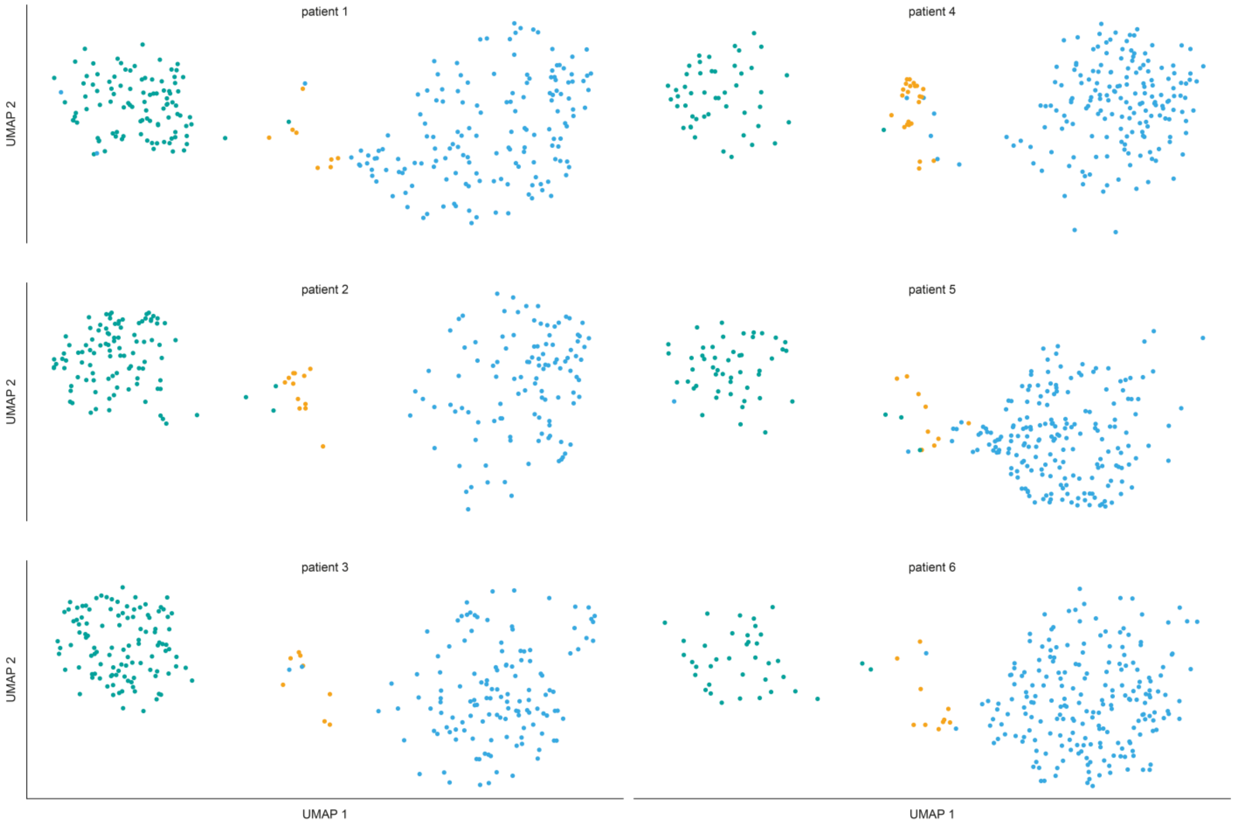

UMAP projection of endometrial cancer B-cell scRNA-seq data per patient and annotated by cluster.

Supplementary Figure 2. Expression of immunoglobulin genes in endometrial tumor-infiltrating B-cells

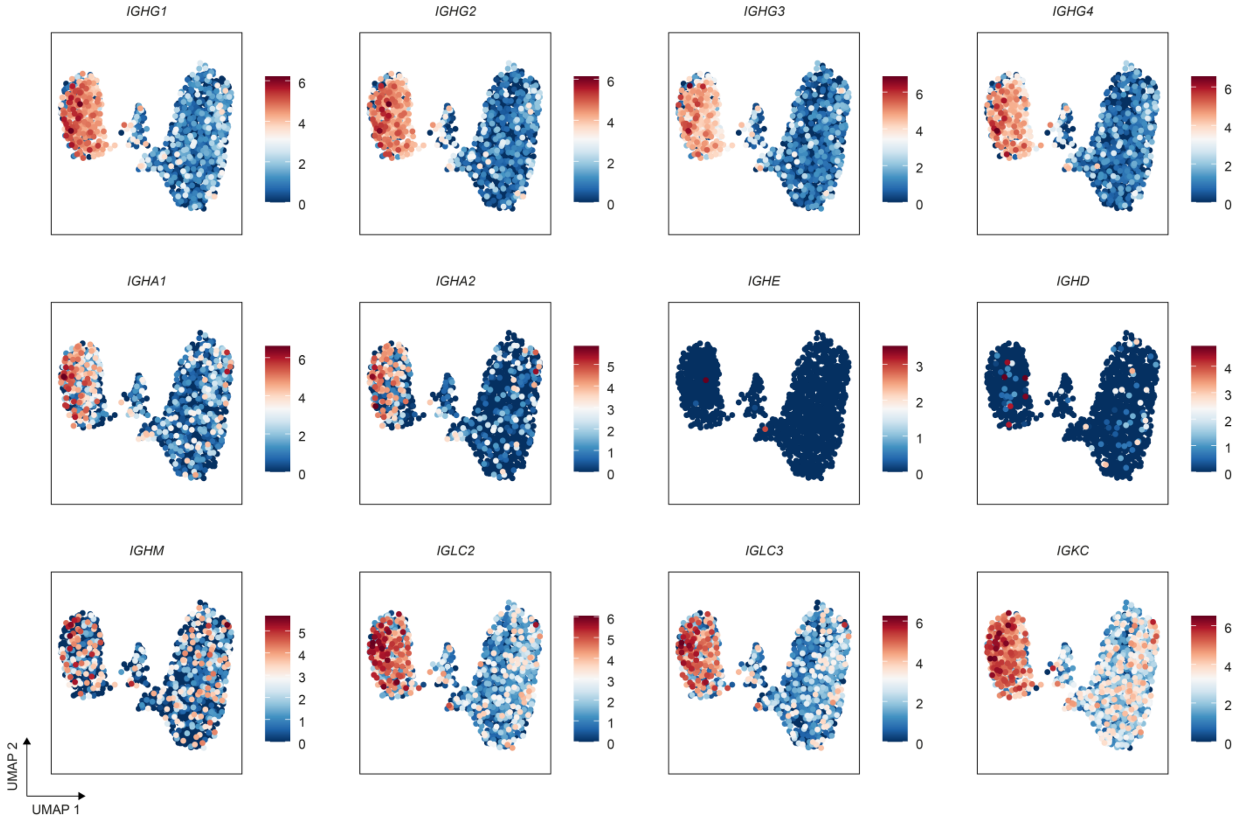

UMAP projection of B-cells with Feature Plots depicting IGHG1-4, IGHA1-2, IGHE, IGHD, IGHM, IGLC2-3 and IGKC expression in single cells.

Supplementary Figure 3. Expression of immunoglobulin genes in endometrial tumor-infiltrating plasma blasts

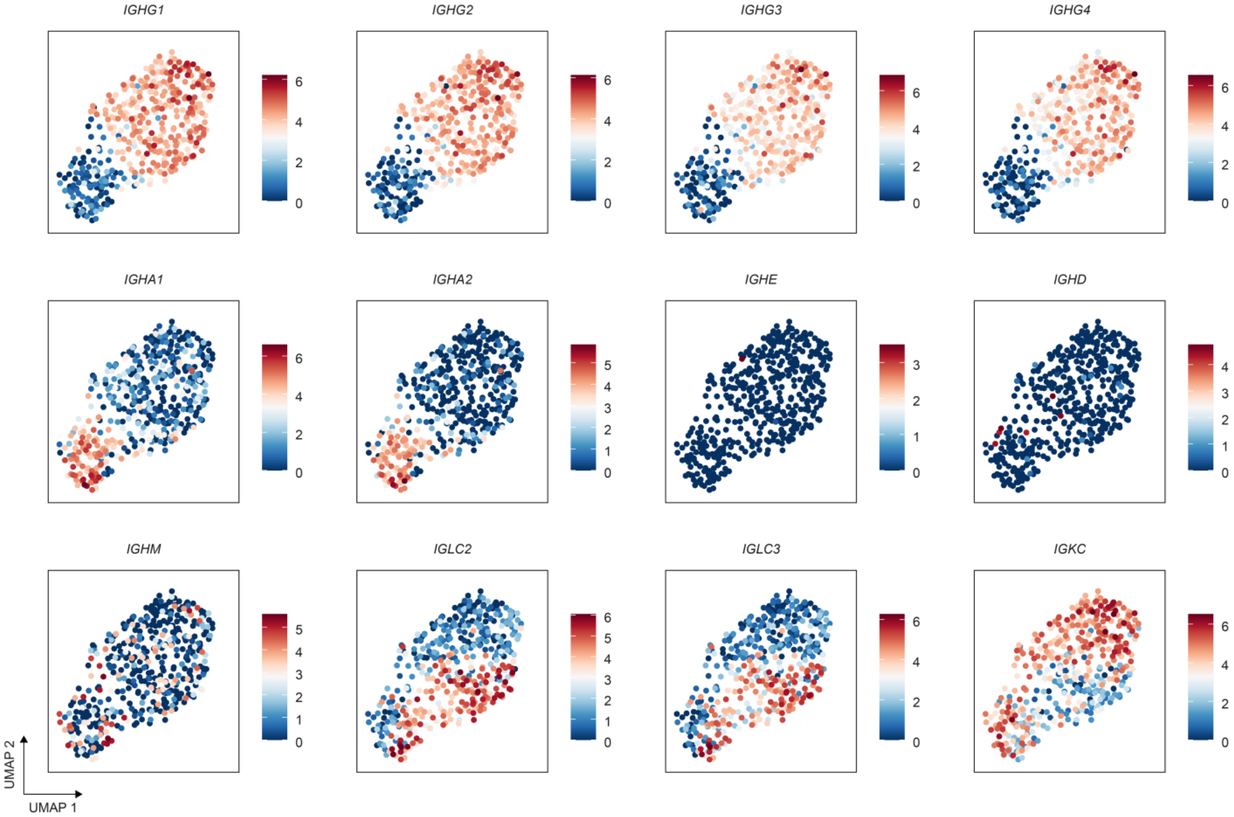

UMAP projection of Cluster 3 plasma blasts with Feature Plots depicting IGHG1-4, IGHA1-2, IGHE, IGHD, IGHM, IGLC2-3 and IGKC expression in single cells.

Supplementary Figure 4. Gene set enrichment analysis of genes differentially expressed between TLS-positive and TLS-negative UCEC TCGA cases

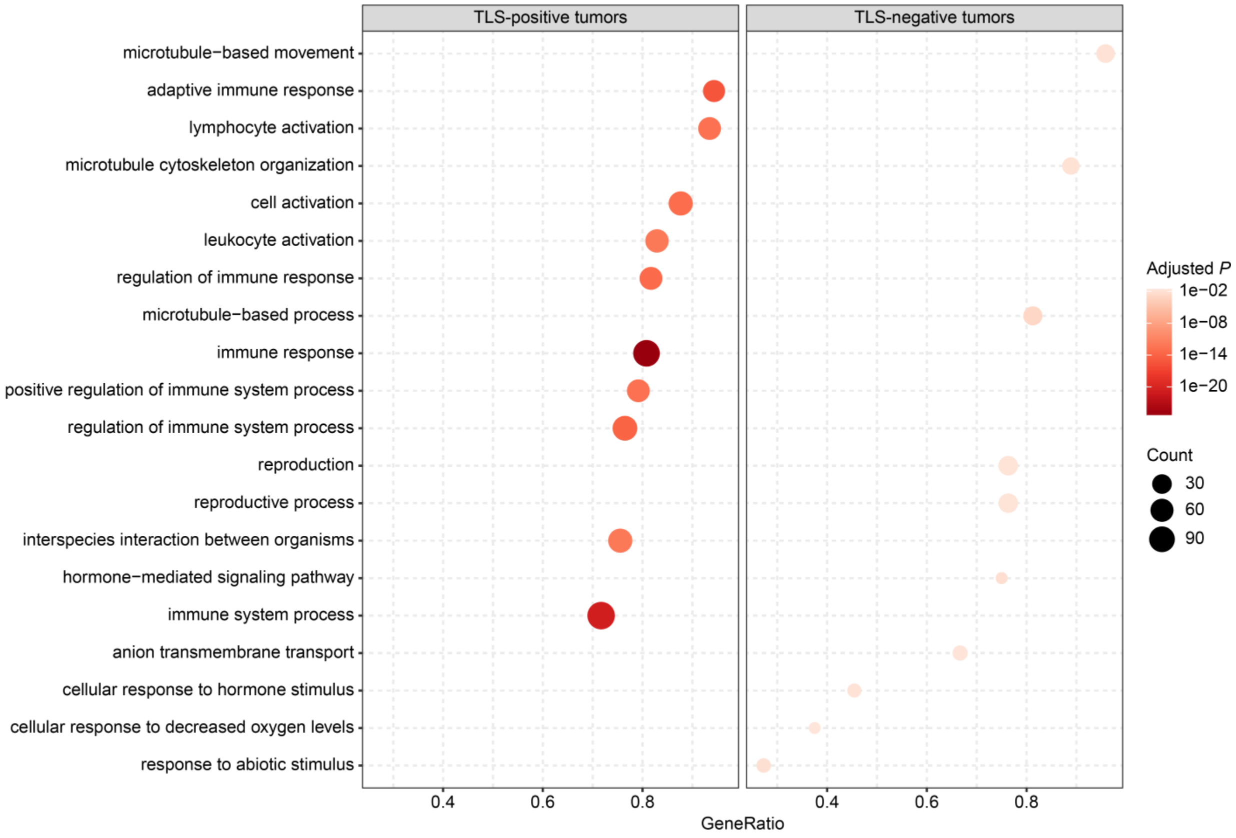

Gene Set Enrichment Analysis was performed using ClusterProfiler (R V4.0.5) with GO terms for biological process. GO terms with a Benjamini–Hochberg FDR <0.05 (two-sided) were selected as significantly different.

Supplementary Figure 5. CONSORT flowchart PORTEC-3

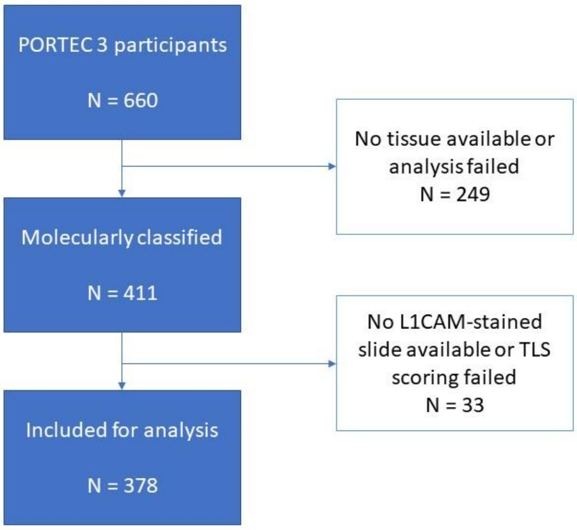

Definition of abbreviations: PORTEC-3 = adjuvant chemoradiotherapy versus radiotherapy alone in women with high-risk endometrial cancer trial; TLS = tertiary lymphoid structure.

Supplementary Tables

Supplementary Table 1. Intra-observer agreement between H&E and L1CAM for the detection of mature TLS

No. of mature TLS by H&E	No. of mature TLS by L1CAM									
	0	1	2	3	4	5	6	8	10	Total
**0**	21	3	0	1	1	0	0	1	0	27
**1**	2	4	2	0	1	1	1	0	0	11
**2**	1	1	1	1	0	1	0	0	0	5
**3**	0	1	0	1	0	0	1	0	0	3
**4**	0	0	0	0	1	0	0	0	0	1
**5**	0	0	0	0	1	0	0	0	0	1
**8**	0	0	0	0	0	0	0	1	0	1
**9**	0	0	0	0	0	0	0	0	1	1
**Total**	24	9	3	3	4	2	2	2	1	50

Concordance considering the counts of the mature TLS: intra-class correlation coefficient: 0.79, 95%CI 0.63-0.89.
Concordance considering presence or absence of mature TLS: kappa 0.64, standard error 0.11.
Definitions of abbreviations: TLS = tertiary lymphoid structures.

Supplementary Table 2. Interobserver agreement between 2 pathologists using L1CAM for the detection of mature TLS

No. of mature TLS by L1CAM according to pathologist 2	No. of mature TLS by L1CAM according to pathologist 1									
	0	1	2	3	4	5	6	8	10	Total
**0**	21	0	0	1	0	0	0	0	0	22
**1**	1	4	0	0	0	0	0	0	0	5
**2**	2	3	2	0	0	0	0	0	0	7
**3**	0	0	1	1	0	0	0	0	0	2
**4**	0	1	0	0	1	0	0	0	0	2
**5**	0	1	0	1	1	0	0	0	0	3
**6**	0	0	0	0	1	1	0	1	0	3
**7**	0	0	0	0	1	1	1	0	0	3
**8**	0	0	0	0	0	0	1	1	0	2
**11**	0	0	0	0	0	0	0	0	1	1
**Total**	24	9	3	3	4	2	2	2	1	50

Concordance considering the counts of the mature TLS: intra-class correlation coefficient: 0.94, 95%CI 0.88-0.97.
Concordance considering presence or absence of mature TLS: kappa 0.84, standard error 0.08.
Definitions of abbreviations: TLS = tertiary lymphoid structures.

Supplementary Table 3. Overview of included and excluded PORTEC-3 participants

Characteristics	Total<br>N (%)	Included<br>N (%)	Excluded<br>N (%)	Difference<br>p-value
No. of patients	660 (100.0)	378 (57.3)	282 (42.7)	
Received treatment				
external beam radiotherapy	333 (50.5)	187 (49.5)	146 (51.8)	0.56
chemoradiotherapy	327 (49.5)	191 (50.5)	136 (48.2)	
Age - median (IQR)	62 (12)	62 (12)	63 (12)	0.21
Histotype and grade				
endometrioid grade 1-2	255 (38.6)	153 (40.5)	102 (36.2)	0.52
endometrioid grade 3	185 (28.0)	104 (27.5)	81 (28.7)	
non-endometrioid	220 (33.3)	121 (32.0)	99 (35.1)	
Stage				
IA	78 (11.8)	50 (13.3)	28 (9.9)	0.53
IB	117 (17.7)	65 (17.2)	52 (18.4)	
II	170 (25.8)	96 (25.4)	74 (26.2)	
III	295 (44.8)	167 (44.3)	128 (45.2)	
Myometrial invasion				
≤50%	239 (36.2)	136 (36.0)	103 (36.5)	0.90
>50%	418 (63.3)	240 (63.5)	178 (63.1)	
unknown	3 (0.5)	2 (0.5)	1 (0.4)	
LVSI				
no	272 (41.2)	139 (36.8)	133 (47.2)	0.007
yes	388 (58.8)	239 (63.2)	149 (52.8)	

The difference between the included and excluded cases were tested using the chi-square test for categorial variables, the Mann-Whitney U test for ordinal variables and non-normally distributed continuous variables. All tests were two-sided with an alpha of 0.05, no adjustments have been made for multiple comparisons.
Definition of abbreviations: IQR = interquartile range; LVSI = lymphovascular space invasion

Supplementary Table 4. Association of TLS with multiple classifying molecular features in high risk endometrial cancer

	**Total**	**TLS: none**	**TLS: ≥1**	**Number of TLS**	
**Molecular features**	**n (%)**	**n (%)**	**n (%)**	**mean**	**range**
*POLE*mut single classifier	31 (100)	15 (48.4)	16 (51.6)	1.48	0-14
MMRd single classifier	117 (100)	94 (80.3)	23 (19.7)	0.74	0-10
p53abn single classifier	83 (100)	78 (94.0)	5 (6.0)	0.19	0-6
NSMP	121 (100)	108 (89.3)	13 (10.7)	0.21	0-5
*POLE*mut, MMRd and p53abn	2 (100)	0 (0.0)	2 (100)	10.50	0-20
*POLE*mut and MMRd	9 (100)	5 (55.6)	4 (44.4)	3.00	0-19
*POLE*mut and p53abn	5 (100)	3 (60.0)	2 (40.0)	2.60	0-9
MMRd and p53abn	10 (100)	4 (40.0)	6 (60.0)	1.00	0-2

According to Léon-Castillo et al. (J Pathol 2020) endometrial cancer with multiple classifying features are assigned to the *POLE*mut group if a pathological *POLE* mutation is present and to the MMRd subgroup in case of MMRd and p53abn.[1]

Among the tumors assigned to the *POLE*mut molecular subgroup (n=47), 31 (66.0%) had a single classifying feature and 16 (34.0%) had multiple classifying features. Among the 31 POLE endometrial cancers with a single classifying feature, 16 (51.6%) had any TLS, among the 16 POLEs with multiple classifying features 8 (50.0%) had any TLS (Fisher exact p = 1.00). Also, the numbers of TLS between these two groups were not significantly different (Mann-Whitney U p = 0.58). All tests were two-sided with an alpha of 0.05, no adjustments have been made for multiple comparisons.

Among the 127 tumors assigned to the MMRd molecular subgroup, TLS were present among 23 of the 117 (19.7%) MMRd endometrial cancer with a single classifying feature; versus TLS presence in 6 out of 10 (60.0%) of those with multiple classifying features (Fisher exact p = 0.009). The numbers of TLS were significantly higher among MMRd/p53abn than MMRd single classifiers (Mann-Whitney U p = 0.013).

Definitions of abbreviations: *POLE*mut = polymerase epsilon mutated endometrial cancer; MMRd = mismatch repair deficient endometrial cancer; p53abn = p53 abnormal endometrial cancer; NSMP = no specific molecular profile endometrial cancer.

Supplementary Table 5. Association of TLS with clinicopathological features and immune cell densities in high risk endometrial cancer

**Characteristics**	**Total**	**TLS: none**	**TLS: ≥1**	**p value**
**All patients**	**378 (100.0)**	**307 (81.2)**	**71 (18.8)**	
Age - median (IQR)	62 (12)	62 (12)	59 (11)	0.13
Histotype and grade				
endometrioid grade 1-2	153 (40.5)	135 (44.0)	18 (25.4)	0.003
endometrioid grade 3	104 (27.5)	74 (24.1)	30 (42.3)	
non-endometrioid	121 (32.0)	98 (31.9)	23 (32.4)	
Stage				
IA	50 (13.2)	37 (12.1)	13 (18.3)	0.15
IB	65 (17.2)	49 (16.0)	16 (22.5)	
II	96 (25.4)	83 (27.0)	13 (18.3)	
III	167 (44.2)	138 (45.0)	29 (40.8)	
Myometrial invasion				
≤50%	136 (36.2)	111 (36.4)	25 (35.2)	0.85
>50%	240 (63.8)	194 (63.6)	46 (64.8)	
LVSI				
no	139 (36.8)	114 (37.1)	25 (35.2)	0.76
yes	239 (63.2)	193 (62.9)	46 (64.8)	
L1CAM expression by the tumour				
negative (none or ≤10%)	273 (72.6)	216 (70.8)	57 (80.3)	0.10
positive (>10%)	103 (27.4)	89 (29.2)	14 (19.7)	
Molecular group				
*POLE*mut	47 (12.4)	23 (7.5)	24 (33.8)	<0.0001
MMRd	127 (33.6)	98 (31.9)	29 (40.8)	
p53abn	83 (22.0)	78 (25.4)	5 (7.0)	
NSMP	121 (32.0)	108 (35.2)	13 (18.3)	
CD8 densities (log2 transformed)				
intraepithelial - median (IQR)	5.5 (3.3)	5.3 (3.1)	7.3 (3.0)	<0.0001
intrastromal - median (IQR)	5.9 (2.9)	5.8 (2.9)	6.9 (2.8)	<0.0001
intratumoral - median (IQR)	6.0 (2.5)	5.8 (2.4)	7.2 (2.8)	<0.0001
CD20 densities (log2 transformed)				
intraepithelial - median (IQR)	3.2 (3.5)	2.9 (3.9)	3.6 (2.7)	0.12
intrastromal - median (IQR)	5.2 (3.9)	4.5 (4.3)	6.4 (3.8)	0.007
intratumoral - median (IQR)	4.2 (3.6)	3.8 (3.7)	5.3 (4.2)	0.024

The difference between the included and excluded cases were tested using the chi-square test for categorial variables, the Mann-Whitney U test for ordinal variables and non-normally distributed continuous variables. All tests were two-sided with an alpha of 0.05, no adjustments have been made for multiple comparisons.
Definitions of abbreviations: TLS = tertiary lymphoid structures; IQR = interquartile range; LVSI = lymphovascular space invasion; *POLE*mut = polymerase epsilon mutated endometrial cancer; MMRd = mismatch repair deficient endometrial cancer; p53abn = p53 abnormal endometrial cancer; NSMP = no specific molecular profile endometrial cancer.

Supplementary Table 6. Association of CD8 densities with clinicopathological features in high risk endometrial cancer

	Intraepithelial		Intrastromal		Intratumoral	
**Characteristics**	**median (IQR)**	**p value**	**median (IQR)**	**p value**	**median (IQR)**	**p value**
Histotype and grade						
endometrioid gr 1-2	5.3 (2.6)	0.13	5.6 (2.5)	0.001	5.7 (2.1)	0.002
endometrioid gr 3	6.1 (3.4)		6.8 (3.2)		6.6 (3.0)	
non-endometrioid	6.0 (4.1)		6.4 (3.5)		6.5 (3.2)	
Stage						
IA	5.8 (4.4)	0.32	6.9 (4.1)	0.088	6.7 (5.2)	0.087
IB	6.2 (3.0)		6.5 (3.6)		6.8 (3.1)	
II	5.2 (3.3)		5.5 (3.2)		5.6 (2.9)	
III	5.7 (3.2)		5.9 (2.5)		6.0 (2.2)	
Myometrial invasion						
≤50%	5.5 (3.4)	0.59	5.9 (3.0)	0.64	5.9 (3.0)	0.55
>50%	5.7 (3.3)		6.0 (2.8)		6.1 (2.4)	
LVSI						
no	5.5 (3.0)	0.54	5.9 (3.3)	0.43	5.8 (2.7)	0.30
yes	5.6 (3.3)		6.0 (2.7)		6.2 (2.4)	
Molecular group						
*POLE*mut	7.2 (3.1)	<0.0001	7.4 (2.8)	<0.0001	7.5 (2.5)	<0.0001
MMRd	6.7 (2.7)		6.6 (2.5)		6.8 (2.7)	
p53abn	4.5 (3.5)		5.0 (3.3)		5.4 (3.2)	
NSMP	4.9 (2.2)		5.3 (2.6)		5.5 (2.0)	

The difference between the included and excluded cases were tested using the chi-square test for categorial variables, the Mann-Whitney U test for ordinal variables and non-normally distributed continuous variables. All tests were two-sided with an alpha of 0.05, no adjustments have been made for multiple comparisons.
Definitions of abbreviations: IQR = interquartile range; LVSI = lymphovascular space invasion; corr = correlation; *POLE*mut = polymerase epsilon mutated endometrial cancer; MMRd = mismatch repair deficient endometrial cancer; p53abn = p53 abnormal endometrial cancer; NSMP = no specific molecular profile endometrial cancer.

Supplementary Table 7. Association of CD20 densities with clinicopathological features in high risk endometrial cancer

	Intraepithelial		Intrastromal		Intratumoral	
**Characteristics**	**median (IQR)**	**p value**	**median (IQR)**	**p value**	**median (IQR)**	**p value**
Histotype and grade						
endometrioid gr 1-2	2.7 (3.6)	0.12	4.3 (3.6)	.002	3.6 (3.2)	0.007
endometrioid gr 3	3.5 (4.0)		6.0 (3.9)		4.9 (4.2)	
non-endometrioid	3.1 (4.2)		5.5 (6.0)		4.1 (5.0)	
Stage						
IA	3.4 (3.2)	0.77	5.8 (4.6)	0.15	4.4 (4.1)	0.48
IB	3.2 (4.0)		5.4 (4.8)		4.0 (4.5)	
II	3.0 (4.2)		4.8 (4.3)		4.0 (3.7)	
III	3.1 (3.8)		4.5 (4.4)		3.8 (3.8)	
Myometrial invasion						
≤50%	3.2 (3.9)	0.69	5.4 (5.1)	0.95	4.2 (3.9)	0.89
>50%	3.1 (3.7)		4.8 (4.3)		3.9 (3.7)	
LVSI						
no	3.0 (4.2)	0.67	5.2 (4.7)	0.48	3.9 (4.0)	0.92
yes	3.2 (3.7)		4.6 (4.2)		4.0 (3.8)	
Molecular group						
*POLE*mut	4.0 (3.6)	<0.009	6.8 (3.3)	<0.0001	5.7 (4.6)	<0.0001
MMRd	3.6 (3.2)		6.1 (3.7)		5.3 (3.6)	
p53abn	2.3 (4.0)		4.0 (5.3)		3.2 (4.5)	
NSMP	2.5 (3.5)		3.8 (3.5)		3.2 (2.9)	

The difference between the included and excluded cases were tested using the chi-square test for categorial variables, the Mann-Whitney U test for ordinal variables and non-normally distributed continuous variables. All tests were two sided with an alpha of 0.05, no adjustments have been made for multiple comparisons.
Definitions of abbreviations: IQR = interquartile range; LVSI = lymphovascular space invasion; corr = correlation; *POLE*mut = polymerase epsilon mutated endometrial cancer; MMRd = mismatch repair deficient endometrial cancer; p53abn = p53 abnormal endometrial cancer; NSMP = no specific molecular profile endometrial cancer.

Supplementary Table 8. Factors associated with TLS presence in high-risk endometrial cancer

	Univariable analysis			Multivariable analysis		
	OR	95% CI	p value	OR	95% CI	p value
Age	0.99	0.96-1.02	0.35	1.01	0.97-1.06	0.57
Histograde						
EEC grade 1-2	reference			reference		
EEC grade 3	3.04	1.59-5.82	0.001	2.01	0.78-5.18	0.15
non-EEC	1.76	0.90-3.44	0.098	1.59	0.55-4.58	0.39
Stage (I-II vs. III)	0.85	0.50-1.43	0.53	1.22	0.56-2.66	0.61
LVSI (no vs. yes)	1.09	0.63-1.86	0.76	0.81	0.37-1.77	0.60
Molecular group						
NSMP	reference			reference		
POLEmut	8.67	3.85-19.51	<0.0001	4.94	1.43-17.05	0.011
MMRd	2.46	1.21-5.00	0.013	1.27	0.49-3.29	0.63
p53abn	0.53	0.18-1.56	0.25	0.31	0.07-1.40	0.13
CD8 density						
intraepithelial	1.38	1.18-1.62	<0.0001			
intrastromal	1.35	1.15-1.58	<0.0001			
intratumoral	1.47	1.23-1.75	<0.0001	1.30	1.04-1.62	0.020
CD20 density						
intraepithelial	1.10	0.98-1.24	0.12			
intrastromal	1.12	1.02-1.23	0.023	0.97	0.84-1.15	0.86
intratumoral	1.12	1.01-1.24	0.033			

Univariable and multivariable regression analysis using Cox proportional hazards models show the association of clinicopathological factors, molecular class and cytotoxic T-cell (CD8) and B-cell (CD20) with presence of tertiary lymphoid structures detected using L1CAM. Covariates were pre-specified according to Léon-Castillo et al. (2020).[2] In the multivariable model, the CD8 and CD20 marker with the strongest association was entered in the multivariable model. -2 Log likelihood = 207.369, Nagelkerke R = 0.239.

Supplementary Table 9. Impact of TLS and immune cell densities on time to recurrence in high-risk endometrial cancer

	Univariable analysis				
	HR	95% CI	p value	C-index (se)	AIC
Recurrence (n=252, 75 events)					
CD8 density					
intraepithelial	0.85	0.78-0.93	0.00049	0.608 (0.034)	780.171
intrastromal	0.87	0.80-0.95	0.0046	0.585 (0.034)	784.093
intratumoral	0.83	0.74-0.93	0.0011	0.597 (0.034)	781.202
CD20 density					
intraepithelial	0.93	0.84-1.03	0.18	0.546 (0.034)	790.026
intrastromal	0.92	0.85-1.00	0.048	0.560 (0.033)	787.936
intratumoral	0.92	0.84-1.01	0.073	0.552 (0.034)	788.614
TLS					
number of TLS	0.62	0.42-0.92	0.017	0.576 (0.018)	778.315
none vs. ≥1	0.25	0.10-0.62	0.0028	0.575 (0.019)	778.397

Univariable Cox proportional hazards models show impact of log2-transformed T-cell (CD8) and B-cell (CD20) densities in the intraepithelial, intrastromal and the intratumoral (intraepithelial + intrastromal) compartments and the presence of tertiary lymphoid structures on time to endometrial recurrence. All tests were two sided with an alpha of 0.05, no adjustments have been made for multiple comparisons. Definition of abbreviations: HR = hazard ratio; CI = confidence interval; C-index - concordance index; se = standard error; AIC = Akaike's Information Criterion; TLS = tertiary lymphoid structure.

Supplementary Table 10. Correlation between immune cell densities and TLS in high-risk endometrial cancer

		CD8			CD20			TLS	
		intra-epithelial	intra-stromal	intra-tumoral	intra-epithelial	intra-stromal	intra-tumoral	number	none vs. ≥1
CD8+	intraepithelial								
	intrastromal	0.848**							
	intratumoral	0.920**	0.955**						
CD20+	intraepithelial	0.390**	0.398**	0.408**					
	intrastromal	0.507**	0.554**	0.541**	0.695**				
	intratumoral	0.493**	0.522**	0.524**	0.797**	0.947**			
TLS	number	0.276**	0.240**	0.272**	0.144*	0.209**	0.191**		
	none vs. ≥1	0.282**	0.246**	0.281**	0.133*	0.198**	0.179**	0.993**	

Spearman's correlation coefficients. Definition of abbreviation: TLS = tertiary lymphoid structures.
* Significance at the <0.05 level (2-tailed permutation test using a T-distribution)
** Significance at the <0.01 level (2-tailed permutation test using a T-distribution)

Supplementary Table 11. Prognostic factors for endometrial cancer-specific survival in high-risk endometrial cancer

	Pathologic model			Molecular model			Molecular-immune model		
	HR	95% CI	p value	HR	95% CI	p value	HR	95% CI	p value
Endometrial cancer-specific survival (n=376, 75 events)									
Age	1.08	1.04-1.11	4.21×10^{-6}	1.06	1.02-1.10	0.00099	1.06	1.03-1.10	0.00075
Adjuvant treatment									
RT	reference			reference			reference		
CTRT	0.68	0.43-1.08	0.11	0.68	0.43-1.07	0.10	0.83	0.54-1.28	0.13
Histograde									
EEC grade 1-2	reference			reference			reference		
EEC grade 3	2.09	1.11-3.92	0.022	1.65	0.82-3.31	0.16	1.95	0.97-3.92	0.061
non-EEC	2.39	1.32-4.35	0.0043	1.22	0.59-2.52	0.59	1.37	0.67-2.82	0.39
Stage (I-II vs. III)	1.79	1.10-2.93	0.020	1.73	1.06-2.80	0.027	1.81	1.11-2.94	0.017
LVSI	1.29	0.75-2.22	0.35	1.29	0.75-2.24	0.36	1.24	0.71-2.17	0.44
Molecular group									
NSMP				reference			reference		
POLEmut				no events			no events		
MMRd				1.41	0.70-2.84	0.34	1.65	0.82-3.31	0.16
p53abn				3.99	1.89-8.40	0.00027	3.52	1.69-7.34	0.00077
TLS							0.15	0.04-0.61	0.0085

Three multivariable Cox proportional hazards models show impact of respectively clinicopathological factors, clinicopathological + molecular factors, and clinicopathological + molecular factors + presence of tertiary lymphoid structures on endometrial cancer-specific survival. Covariates were pre-specified according to Léon-Castillo et al. (2020).[2] The addition of the molecular classifier to the pathologic model was associated with an improvement in model fit evidenced by: (i) reduction in Akaike's information criterion (AIC) 828.372 vs. 797.269, (ii) increase in model concordance (C index 0.719 vs. 0.777 and (iii) likelihood ratio test for comparison of nested models $p = 4.38 \times 10^{-8}$. Likewise, the addition of TLS presence improved model fit: (i) AIC 797.269 vs. 786.669, (ii) C index 0.777 vs. 0.796 (iii) likelihood ratio test for nested models p = 0.00039. All tests were two-sided with an alpha of 0.05, no adjustments have been made for multiple comparisons. Definition of abbreviations: HR = hazard ratio; CI = confidence interval; RT = external beam radiotherapy; CTRT = chemoradiotherapy; EEC = endometrioid endometrial cancer; LVSI = lymphovascular space invasion; NSMP = no specific molecular profile; *POLE*mut = pathogenic polymerase epsilon mutation; MMRd = mismatch repair deficient; p53abn = p53 abnormal; TLS = tertiary lymphoid structure

Supplementary Table 12. Sensitivity analysis: prognostic impact of immuno-biomarkers for recurrence of high-risk endometrial cancer

	TLS (none vs. ≥1)			Intraepithelial CD8+ density			Intrastromal CD20+ density		
	HR	95% CI	p value	HR	95% CI	p value	HR	95% CI	p value
*Recurrence (n=252, 75 events)**									
Age	1.03	1.00-1.06	0.045	1.03	1.0-1.06	0.059	1.03	1.00-1.07	0.042
Adjuvant treatment									
RT	reference			reference			reference		
CTRT	0.54	0.34-0.86	0.010	0.52	0.33-0.99	0.0082	0.54	0.34-0.87	0.011
Histograde									
EEC grade 1-2	reference			reference			reference		
EEC grade 3	0.84	0.40-1.76	0.64	0.90	0.44-1.86	0.77	0.80	0.38-1.70	0.57
non-EEC	0.60	0.28-1.32	0.21	0.60	0.27-1.36	0.22	0.57	1.08-3.02	0.17
Stage (I-II vs. III)	1.82	1.09-3.04	0.022	1.79	1.07-3.00	0.027	1.81	1.08-3.02	0.023
LVSI	1.10	0.63-1.90	0.74	1.13	0.66-1.95	0.66	1.12	0.64-1.94	0.69
Molecular group									
NSMP	reference			reference			reference		
POLEmut	no events			no events			no events		
MMRd	1.32	0.72-2.40	0.37	1.50	0.80-2.82	0.21	1.25	0.68-2.29	0.48
p53abn	0.47	2.08-10.61	0.00020	5.60	2.42-12.96	$5.90x10^{-5}$	5.06	2.21-11.57	0.00012
Immunological factor	0.39	0.16-0.99	0.048	0.89	0.80-0.99	0.029	0.98	0.91-1.07	0.69
C-index (se)	0.745 (0.026)			0.736 (0.027)			0.731 (0.027)		
AIC	747.391			747.731			752.272		

* Only cases with complete data on TLS, CD8 and CD20 were included for analysis.

Three multivariable Cox proportional hazards models show impact of clinicopathological factors, molecular class and 3 immunological factors on time to recurrence. Covariates were pre-specified according to Léon-Castillo et al. (2020).[2] All tests were two-sided with an alpha of 0.05, no adjustments have been made for multiple comparisons. Definition of abbreviations: HR = hazard ratio; CI = confidence interval; RT = external beam radiotherapy; CTRT = chemoradiotherapy; EEC = endometrioid endometrial cancer; LVSI = lymphovascular space invasion; NSMP = no specific molecular profile; *POLE*mut = pathogenic polymerase epsilon mutation; MMRd = mismatch repair deficient; p53abn = p53 abnormal; TLS = tertiary lymphoid structure

Supplementary Table 13. Biomarker analysis performed and reported in this study

Analysis	Objective	Endpoint	Population	Methods	Reported
Prognostic impact TLS	Primary	TTR, CCS	PORTEC-3	KM analysis log rank test, MV regression using Cox proportional hazards models	Main text, Table 1, Figure 3B-C, Figure 4, Table S8
Relation TLS with molecular subgroups and CD8	Secondary	Incidence	PORTEC-3	Quantification of TLS on L1CAm stained whole slides, descriptive statistics, supervised hierarchical cluster analysis	Main text, Figure 3A, Tables S3-7
Prognostic impact CD8 and CD20	Sensitivity	TTR	PORTEC-3	MV regression using Cox proportional hazards models	Main text, Table S10
Prognostic impact TLS by molecular group	Exploratory	TTR	PORTEC-3	KM analysis, log rank test	Main text, Figure 3D-E

Supplementary Notes

Members of the PORTEC 3 study group

Department of Radiation Oncology, S M de Boer, R A Nout, C L Creutzberg, department of Pathology T Bosse, V T H B M Smit, and Department of Medical Statistics H Putter, Leiden University Medical Center, Leiden, Netherlands. Department of Clinical Oncology M E Powell and Department of Cellular Pathology N Singh, Barts Health NHS Trust, London, UK; Division of Cancer Medicine L Mileshkin and Division of Radiation Oncology P Khaw, Peter MacCallum Cancer Centre, Melbourne, VIC, Australia L Mileshkin; Department of Surgical Sciences, Gynecologic Oncology, Città della Salute and S Anna Hospital, University of Turin, Turin, Italy D Katsaros; CCTG, Department of Obstetrics and Gynaecology, University of Sherbrooke, Sherbrooke, QC, Canada P Bessette; Department of Radiotherapy, Institut Gustave Roussy, Villejuif, France C Haie-Meder MD; Department of Medical Oncology, RadboudUMC, Nijmegen, Netherlands P B Ottevanger; Cancer Research UK, London, UK: J A Ledermann, A Feeney; UCL Cancer Trials Centre, UCL Cancer Institute, London, UK J A Ledermann, A Feeney; Division of Radiation Oncology, ASST-Lecco, Ospedale AManzoni, Lecco, Italy A Colombo, R D'Amico; CCTG, Radiation Medicine Program, Princess Margaret Cancer Centre, Toronto, ON, Canada A Fyles; Department of Radiotherapy, Centre Hospitalier Régional Universitaire de Besançon, Besançon, France M-H Baron; Department of Radiation Oncology, University Medical Center Utrecht, Netherlands I M Jürgenliemk-Schulz; Institute of Cancer Sciences, University of Manchester, Manchester, UK H C Kitchener MD; Department of Gynaecologic Oncology, University Medical Center Groningen, University of Groningen, Groningen, Netherlands H W Nijman MD; Department of Pathology, Central Manchester Hospitals NHS Foundation Trust, Manchester Royal Infirmary, Manchester, UK G Wilson; Department of Radiation Oncology, Auckland City Hospital, Auckland, New Zealand S Brooks; Division of Pathology and Laboratory Medicine, European Institute of Pathology, Milan, Italy S Carinelli; Department of Gynaecologic Oncology, Hôpital Notre-Dame de Montreal, Montreal, QC, Canada D Provencher; Department of Radiation Oncology, Centre Henri Becquerel, Rouen, France C Hanzen; Department of Radiation Oncology (MAASTRO), GROW School for Oncology and Developmental Biology, Maastricht University Medical Centre, Maastricht, Netherlands L C H W Lutgens; Radiation Oncology Network, CPMCC Westmead, Westmead, NSW, Australia V Do; and Comprehensive Cancer Center Netherlands, Leiden,
Netherlands K W Verhoeven-Adema

Members of the TransPORTEC consortium

Department of Radiation Oncology N Horeweg, S M de Boer, C L Creutzberg, department of Pathology T Bosse, V T H B M Smit, and Department of Medical Oncology J Kroep, Leiden University Medical Center, Leiden, The Netherlands; Department of Radiation Oncology, Erasmus MC Cancer Center, Rotterdam, The Netherlands R A Nout; Department of Gynaecologic Oncology, University Medical Center Groningen, The Netherlands H W Nijman, de Bruyn M. Department of Clinical Oncology M E Powell and Department of Cellular Pathology N Singh, Barts Health NHS Trust, London, UK; Manchester Academic Health Science Centre, St Mary's Hospital, Obstetrics and Gynaecology, Manchester, UK H C Kitchener, E Crosbie, Edmondson R; Oxford University Hospitals NHS Foundation Trust, Oxford NIHR Comprehensive Biomedical Research Centre, Oxford, UK D N Church. Department of Radiotherapy, Institut Gustave Roussy, Villejuif, France A Leary, Division of Cancer Medicine, Peter MacCallum Cancer Centre, Melbourne, VIC, Australia L Mileshkin; School of Biomedical Sciences, Faculty of Health, Queensland University of Technology, Translational Research Institute, Princess Alexandra Hospital Campus, Brisbane, Australia P M Pollock. Odette Cancer Center, Sunnybrook Health Sciences Centre, Medical Oncology,
Toronto, Canada H MacKay.

Supplementary References

1. Leon-Castillo A, Gilvazquez E, Nout R, Smit VT, McAlpine JN, McConechy M, Kommoss S, Brucker SY, Carlson JW, Epstein E, Rau TT, Soslow RA, Ganesan R, Matias-Guiu X, Oliva E, Harrison BT, Church DN, Gilks CB, Bosse T. Clinicopathological and molecular characterisation of 'multiple-classifier' endometrial carcinomas. *J Pathol* 250(3), 312-322 (2020).

2. Leon-Castillo A, de Boer SM, Powell ME, Mileshkin LR, Mackay HJ, Leary A, Nijman HW, Singh N, Pollock PM, Bessette P, Fyles A, Haie-Meder C, Smit V, Edmondson RJ, Putter H, Kitchener HC, Crosbie EJ, de Bruyn M, Nout RA, Horeweg N, Creutzberg CL, Bosse T, Trans Pc. Molecular Classification of the PORTEC-3 Trial for High-Risk Endometrial Cancer: Impact on Prognosis and Benefit From Adjuvant Therapy. *J Clin Oncol* 38, 3388-3397 (2020).
