# Supplementary material for: Tertiary lymphoid structures critical for prognosis in endometrial cancer patients
Source: Nat Commun. 2022 Mar 16;13:1373. doi: 10.1038/s41467-022-29040-x (PMC8927106; doi:10.1038/s41467-022-29040-x)
Supplement: Supplementary file 2 — Reporting Summary [file 41467_2022_29040_MOESM2_ESM.pdf]

nature portfolio

Corresponding author(s): Dr. Nanda Horeweg

Last updated by author(s): Jan 19, 2022

Reporting Summary

Nature Portfolio wishes to improve the reproducibility of the work that we publish. This form provides structure for consistency and transparency in reporting. For further information on Nature Portfolio policies, see our Editorial Policies and the Editorial Policy Checklist.

Statistics

For all statistical analyses, confirm that the following items are present in the figure legend, table legend, main text, or Methods section.

n/a	Confirmed	
☐	☒	The exact sample size (n) for each experimental group/condition, given as a discrete number and unit of measurement
☐	☒	A statement on whether measurements were taken from distinct samples or whether the same sample was measured repeatedly
☐	☒	The statistical test(s) used AND whether they are one- or two-sided<br>*Only common tests should be described solely by name; describe more complex techniques in the Methods section.*
☐	☒	A description of all covariates tested
☐	☒	A description of any assumptions or corrections, such as tests of normality and adjustment for multiple comparisons
☐	☒	A full description of the statistical parameters including central tendency (e.g. means) or other basic estimates (e.g. regression coefficient) AND variation (e.g. standard deviation) or associated estimates of uncertainty (e.g. confidence intervals)
☐	☒	For null hypothesis testing, the test statistic (e.g. F, t, r) with confidence intervals, effect sizes, degrees of freedom and P value noted<br>*Give P values as exact values whenever suitable.*
☒	☐	For Bayesian analysis, information on the choice of priors and Markov chain Monte Carlo settings
☐	☒	For hierarchical and complex designs, identification of the appropriate level for tests and full reporting of outcomes
☐	☒	Estimates of effect sizes (e.g. Cohen's d, Pearson's r), indicating how they were calculated

Our web collection on statistics for biologists contains articles on many of the points above.

Software and code

Policy information about availability of computer code

Data collection	No software was used
Data analysis	SPSS version 25. R version 3.6.3. STAR version 2.5.2. No custom code was used.

For manuscripts utilizing custom algorithms or software that are central to the research but not yet described in published literature, software must be made available to editors and reviewers. We strongly encourage code deposition in a community repository (e.g. GitHub). See the Nature Portfolio guidelines for submitting code & software for further information.

Data

Policy information about availability of data

All manuscripts must include a data availability statement. This statement should provide the following information, where applicable:

- Accession codes, unique identifiers, or web links for publicly available datasets
- A description of any restrictions on data availability
- For clinical datasets or third party data, please ensure that the statement adheres to our policy

Primary datasets: Tthe raw scRNA-seq data generated in this study is available at NCBI Gene Expression Omnibus (GEO) via https://www.ncbi.nlm.nih.gov/geo/query/acc.cgi?acc=GSE180091 under registration number GSE180091, and in the Source Data Files published alongside this article.
RPrimary datasets: the raw scRNA-seq data generated in this study is available at NCBI Gene Expression Omnibus (GEO) via https://www.ncbi.nlm.nih.gov/geo/query/acc.cgi?acc=GSE180091 under registration number GSE180091, and in the Source Data Files published alongside this article. Source data are provided with this paper.

Reference datasets: The study protocol and other documentation of the PORTEC-3 trial are publicly available at http://msbi.nl/portec3. The tumour material and

datasets generated during and/or analysed during the current study of the PORTEC-3 trial participants are not publicly available due to restrictions by privacy laws. Data and tumour material are currently available to the members of the international TransPORTEC consortium, and the consortium is open for requests for sharing of the data and material after receipt and evaluation of a scientific proposal. Requests should be addressed to the corresponding author within 15 years from the date of publication. Depending on the specific research proposal, the TransPORTEC consortium will determine when, for how long, for which specific purposes, and under which conditions the requested data can be made available, subject to ethical consent.
The TCGA-UCEC data used in this study are available in the National Cancer Institute database and publicly accessible via the GDC data portal [https://portal.gdc.cancer.gov/projects/TCGA-UCEC].

Field-specific reporting

Please select the one below that is the best fit for your research. If you are not sure, read the appropriate sections before making your selection.

- [x] Life sciences
- [ ] Behavioural & social sciences
- [ ] Ecological, evolutionary & environmental sciences

For a reference copy of the document with all sections, see nature.com/documents/nr-reporting-summary-flat.pdf

Life sciences study design

All studies must disclose on these points even when the disclosure is negative.

Sample size	This is not a clinical trial, but a study that assesses the independent prognostic value of a new biomarker.<br>The sample size is in this case determined by the number of recurrence events required to perform multivariable analysis including all widely accepted prognostic factors (such as histology, grade, stage, LVSI, molecular class) and the new biomarker. In this case at least 100 patients with a recurrence were required. The dataset of the PORTEC 3 trial is of very high quality and has more than 100 patients with a recurrence.
Data exclusions	Exclusion criteria were predefined and kept as limited as possible.<br>We only required the cases to be molecularly classified and to have a successful assessment of the new biomarker
Replication	Model validation was performed by analysis of discrimination and indices of optimism determined by means of model fitting to 1000 bootstrap resamples.
Randomization	Again this is not a clinical trial, but we do use data of the international randomized PORTEC 3 trial. Eligible patients were randomly allocated (1:1) to chemoradiotherapy or radiotherapy alone. Treatment was allocated with a biased-coin minimisation procedure, ensuring balance overall and within each stratum of the stratification factors (participating centre, lymphadenectomy, stage of cancer, and histological type).
Blinding	Participants and investigators were not masked to treatment allocation because this is not possible (those in the experimental are had chemotherapy in addition to radiotherapy).<br>In the interobserver substudy that was added in the revised version of the manuscript, the two pathologists were blinded for all clinicopathological and molecular characteristics of the cases.

Reporting for specific materials, systems and methods

We require information from authors about some types of materials, experimental systems and methods used in many studies. Here, indicate whether each material, system or method listed is relevant to your study. If you are not sure if a list item applies to your research, read the appropriate section before selecting a response.

Materials & experimental systems

n/a	Involved in the study	
[ ]	[x]	Antibodies
[x]	[ ]	Eukaryotic cell lines
[x]	[ ]	Palaeontology and archaeology
[x]	[ ]	Animals and other organisms
[ ]	[x]	Human research participants
[ ]	[x]	Clinical data
[x]	[ ]	Dual use research of concern

Methods

n/a	Involved in the study	
[x]	[ ]	ChIP-seq
[x]	[ ]	Flow cytometry
[x]	[ ]	MRI-based neuroimaging

Antibodies

Antibodies used	primary monoclonal antibodies against L1CAM (CD171; clone 14.10; 1:500; SIG-3911; Convance Inc.)<br>mouse anti-human CD8 (3 mg/L, clone C8/144B, GA62361–2, DAKO, Agilent Technologies)<br>mouse anti-human CD20 (0.63 mg/L; clone L26, catalog number M0755, Dako)
Validation	L1CAM: the antibody has been validated for use in humans, details are provided at the website of the manufacturer: https://

www.biolegend.com/en-gb/products/purified-anti-cd171-l1-antibody-11299?Clone=14.10#productCertificate
CD8: the antibody has been validated for use in human diagnostics, details are provided at the website of the manufacturer: https://www.agilent.com/cs/library/packageinsert/public/P02239EFG_05.pdf
CD20: the antibody has been validated for use in humans, details are provided at the website of the manufacturer: https://www.agilent.com/en/product/immunohistochemistry/antibodies-controls/primary-antibodies/cd20cy-(concentrate)-76520

Human research participants

Policy information about studies involving human research participants

Population characteristics	Female patients aged a median of 62 years (IQR 12 years) with high-risk endometrial cancer. The cancers were diagnosed at stage IA (11.8%), IB (17.7%), II (25.8%) and III (44.8%). 38.6% of the endometrial cancers were low grade endometrioid cancers, 28.0% high grade endometrioid cancers and 33.3% non-endometrioid endometrial cancers. Myometrial invasion was found in 63.3% and lymphovascular space invasion was present in 58.8%. All patients were treated with radical hystectomy before inclusion. Thereafter patients were randomized between chemoradiation and radiotherapy only.
Recruitment	Patients were recruited at at 103 centres (oncology centres, university hospitals, regional hospitals, or radiation oncology centres with referrals from regional hospitals) in six clinical trial groups collaborating in the Gynaecological Cancer Intergroup. Participating groups were the National Cancer Research Institute (UK), Australia and New Zealand Gynaecologic Oncology Group, Mario Negri Gynaecologic Oncology Group (Italy), Canadian Cancer Trials Group and Fedegyn (France). It is unlikely that any selection bias affects the results of this study.
Ethics oversight	Written informed consent has been obtained from all patients. The study protocol was approved by the Ethics Committees of all participating groups and is available online at: http://msbi.nl/portec3

Note that full information on the approval of the study protocol must also be provided in the manuscript.

Clinical data

Policy information about clinical studies

All manuscripts should comply with the ICMJEguidelines for publication of clinical research and a completedCONSORT checklist must be included with all submissions.

Clinical trial registration	ISRCTN14387080, NCT00411138
Study protocol	The protocol of the trial data that we used for our study can be found at http://msbi.nl/portec3
Data collection	PORTEC-3 was an open-label, randomised, phase 3 trial at 103 centres in six clinical trial groups collaborating in the Gynaecological Cancer Intergroup. Participating groups were the National Cancer Research Institute (NCRI; UK), Australia and New Zealand Gynaecologic Oncology Group, Mario Negri Gynaecologic Oncology Group (MaNGO; Italy), Canadian Cancer Trials Group (CCTG; Canada), and Fedegyn (France). Patients were recruited from 2006-2013. Data collection still continues today. For this study, data collected up to 5 years after randomization was used.
Outcomes	The outcomes overall recurrence and EC-specific survival were predefined, this data was prospectively collected in the PORTEC 3 trial.
