# Supplementary material for: Tertiary lymphoid structures critical for prognosis in endometrial cancer patients
Source: Nat Commun. 2022 Mar 16;13:1373. doi: 10.1038/s41467-022-29040-x (PMC8927106; doi:10.1038/s41467-022-29040-x)
Supplement: Supplementary file 4 — Description of Additional Supplementary Files [file 41467_2022_29040_MOESM4_ESM.pdf]

Title: Supplementary Data 1. Genes differentially expressed between EC B-TIL clusters
Description: Results of the differential gene expression analysis comparing three clusters of tumor-infiltrating B-cells in endometrial cancer: plasmablasts, naïve B-cells and germinal center/cycling B-cells.

Title: Supplementary Data 2. Genes differentially expressed between IgG and IgA B-TIL clusters
Description: Results of the differential gene expression analysis comparing two clusters of tumor-infiltrating B-cells in endometrial cancer: IgA and IgG.

Title: Supplementary Data 3. Genes differentially expressed between TLS-positive and TLS-negative UCEC TCGA cases
Description: Results of the differential gene expression analysis comparing endometrial cancer patients included in the Uterine Corpus Endometrial Carcinoma cohort of The Cancer Genome Atlas with and without tertiary lymphoid structures.

Title: Supplementary Data 4. GSEA for DE genes between TLS-positive and TLS-negative UCEC TCGA cases
Description: Results of for gene set enrichment analysis comparing endometrial cancer patients included in the Uterine Corpus Endometrial Carcinoma cohort of The Cancer Genome Atlas with and without tertiary lymphoid structures.
